# Prevalence of discospondylitis and association with congenital vertebral body malformations in English and French bulldogs

**DOI:** 10.1111/jvim.17209

**Published:** 2024-10-03

**Authors:** Nick Grapes, Simon Bertram, Rita Gonçalves, Steven De Decker

**Affiliations:** ^1^ Neurology and Neurosurgery Davies Veterinary Specialists Higham Gobion United Kingdom; ^2^ Neurology and Neurosurgery Cave Veterinary Specialists Wellington United Kingdom; ^3^ Department of Veterinary Science, Small Animal Teaching Hospital University of Liverpool Chesire United Kingdom; ^4^ Department of Clinical Science & Services Royal Veterinary College Hatfield United Kingdom

**Keywords:** diskospondylitis, hemivertebra, neurology, small animal

## Abstract

**Background:**

Limited current information exists regarding discospondylitis within breeds commonly affected by congenital vertebral body malformations.

**Hypothesis/Objectives:**

Report the prevalence of discospondylitis in English and French bulldogs and investigate for possible associations of discospondylitis with congenital vertebral body malformations.

**Animals:**

108 client‐owned dogs.

**Methods:**

Retrospective multi‐institutional study between June 2010 and 2020. Cases with a diagnosis of discospondylitis on computed tomography (CT) or magnetic resonance imaging (MRI) and complete medical records included. Signalment, discospondylitis location, presence of congenital vertebral body malformations, and the site of maximal kyphosis were recorded.

**Results:**

The prevalence of discospondylitis was 3.4 (1.6‐6.7, 95% confidence interval [CI]) times higher in French bulldogs (*P* < .001) and 4.3 (1.7‐9.8, 95% CI) times higher in English bulldogs (<.001), compared with the overall hospital cohort. One or more vertebral malformations were present in 12 French bulldogs (92.3%), 6 English bulldogs (75.0%), and 1 “other” breed dog (1.1%). Discospondylitis was diagnosed adjacent to congenital vertebral body malformations in 12 (80%) intervertebral discs in French bulldogs and 5 (50%) intervertebral discs in English bulldogs. The median age at presentation was significantly younger in French bulldogs (1.1 years; range, 0.5‐9.2 years) and English bulldogs (1.0 years; range, 0.4‐7.0 years), compared with “other” breed dogs (7.3 years; range, 0.3‐14.0 years; both *P* < .001).

**Conclusions and Clinical Importance:**

Congenital vertebral body malformations were frequently associated with discospondylitis in French and English bulldogs, with clinical signs commonly encountered at a significantly younger age.

**Abbreviations**
CSF: cerebrospinal fluid
CVM: congenital vertebral body malformation
CT: computed tomography
IVD: intervertebral disc
MRI: magnetic resonance imaging

## 1 INTRODUCTION

Discospondylitis is characterized by infection, most commonly bacterial, of an intervertebral disc (IVD) and the associated vertebral endplates.^1^ Infection is most commonly via hematogenous spread from a remote site within in the body; however, other causes include trauma and tracking foreign bodies.^2^, ^3^, ^4^ Multiple bacterial species have been isolated, with staphylococcus being the most frequently identified in many studies.^5^ Fungal and algal causes have also been demonstrated.^6^, ^7^, ^8^ Clinical signs might include spinal hyperesthesia, paresis, ataxia, exercise intolerance, inappetence and pyrexia, in a minority of cases.^2^, ^5^ Diagnosis might be achieved radiographically; however, the use of computed tomography (CT) or magnetic resonance imaging (MRI) could allow for diagnosis earlier in the course of disease.^9^, ^10^, ^11^ Treatment typically involves long courses of antibiotics with no definitive therapeutic recommendation.^12^

Congenital vertebral body malformations are commonly identified in “screw‐tailed” brachycephalic breeds.^13^, ^14^, ^15^, ^16^, ^17^ Selective breeding for a curled tail is attributed to the development of vertebral malformations within other regions of the vertebral column, with truncation of the *DISHEVELED 2* gene having been implicated.^18^, ^19^ Vertebral body malformations are frequently identified incidentally, with a prevalence of 78.9% to 100% and 73.2% to 83.4% in neurologically normal French and English bulldogs, respectively.^13^, ^20^, ^21^ The degree of kyphosis can be quantified by measuring the Cobb angle with values in excess of 35° being associated with a higher likelihood of clinical signs in multiple studies.^16^, ^20^, ^21^, ^22^ Conversely, the presence of a kyphosis exceeding 35° can also be observed in the absence of concurrent neurological signs.^23^, ^24^ Although vertebral body malformations should only rarely be considered the primary cause of neurological signs in French and English bulldogs, their presence has been associated with altered gait variables and identified as a potential contributing factor for the development of other conditions.^25^ Vertebral body malformations have been associated with early degeneration of the adjacent IVDs, almost 2 times the odds of having a thoracolumbar IVD extrusion and a greater tendency for IVD extrusions to occur at a more caudal location within the thoracolumbar vertebral column.^23^, ^25^, ^26^ However, it remains unknown whether there is an association between vertebral body malformations and other IVD‐associated spinal conditions, such as discospondylitis.

Our aim was to report the prevalence of discospondylitis in French and English bulldogs and investigate for evidence of association between discospondylitis and congenital vertebral body malformations. It was hypothesized that discospondylitis would be more prevalent in French and English bulldogs with predisposition to IVDs adjacent to congenital vertebral body malformations.

## 2 MATERIALS AND METHODS

Ethical approval for this retrospective study was granted by the Royal Veterinary College Social Sciences Research Ethical Review Board. The digital medical databases of the Small Animal Teaching Hospitals of the Royal Veterinary College and University of Liverpool, were searched to retrieve the records of all dogs diagnosed with discospondylitis between June 2010 and June 2020. The search terms of “discospondylitis” and “diskospondylitis” were used to identify patient records. Dogs were required to have undergone a general physical examination and complete neurological examination with advanced imaging (CT or MRI) performed to obtain a radiographic diagnosis of discospondylitis. Neurological examinations were performed by a board‐certified neurologist or a neurology resident under the direct supervision of a board‐certified neurologist. Cases with incomplete medical records or imaging studies were excluded from the study. The diagnostic tests performed for each patient were decided upon by the attending board‐certified neurologist and included: CT, MRI, cerebrospinal fluid (CSF) analysis, blood tests including hematology and biochemistry, infectious disease serology and bacterial culture of urine, blood, or needle aspirates of affected IVDs. CT was performed with a 16‐slice (PQ 500, Universal systems, Solon; GE Healthcare), 80‐slice (Aquilion Prime, Canon Medical Systems) or 320‐slice Helical scanner (Aquilion ONE Genesis Edition, Canon Medical Systems), whereas MRI was performed with a high field unit (1.5 T, Intera or Ingenia CX; Philips Healthcare, the Netherlands). All imaging studies were reviewed by a board‐certified neurologist, radiologist, or both. A diagnosis of discospondylitis was reached using a combination of compatible clinical signs and previously defined changes to the vertebral endplates, IVD, and surrounding soft tissues with advanced imaging (MRI/CT).^9^, ^11^ For the purposes of the study, all imaging studies were retrospectively reviewed for the presence of vertebral body malformations and kyphosis. Congenital vertebral body malformations were identified. A subjective assessment of the IVD or discs located at the location of the greatest deviation of the vertebral column was made to determine the location of maximal kyphosis.

For all cases within the study cohort, the following data were collected from medical records: age, breed, sex, neuter status, weight, breed, IVD space or spaces affected, presence of vertebral body malformation including the type and location, and the location within the vertebral column of maximal kyphosis. Breed was further categorized as either French bulldog, English bulldog or “other” encompassing dogs of all remaining breeds, including crossbreeds. The presence of discospondylitis adjacent to vertebral body malformations or the site of maximal kyphosis was recorded as a binary categorical variable. Discospondylitis location was grouped as cervical (C1‐T1), thoracolumbar (T1‐L7), lumbosacral (L7‐S1) for further analysis. In dogs with discospondylitis suspected at multiple IVDs, each affected IVD was recorded individually.

Statistical analysis was performed using a standard statistical software package (SPSS V.28.0.0.0; IBM). To determine over‐ or under representation of each breed, the clinically affected dogs were compared to the overall hospital cohort for the study period and analyzed via cross‐tabulation and chi‐square analysis. The association between non‐normally distributed continuous variables (age) with breed was assessed using a Kruskal‐Wallis test with post hoc pairwise comparison. Analysis of categorical data were performed using cross‐tabulations with chi‐squared and Fisher's exact tests. Non‐normally distributed continuous data are presented as median (range) while normally distributed data are presented as mean (SD). For all tests *P* values <.05 were considered significant.

## 3 RESULTS

Discospondylitis was diagnosed in 108 dogs, equating to 0.14% (108/ 78 066) of the total dogs presented to both institutions during the study window. Of these, 13 were French bulldogs (0.48% of 2690 French bulldogs presented), and 8 were English bulldogs (0.60% of 1330 English bulldogs presented), whereas the remaining 87 (0.12% of 74 046 other breeds presented) were “other” breeds, including crossbreed dogs. No pugs (0% of 1872 dogs) were diagnosed with discospondylitis during the study period. Of the 87 “other” breed dogs, crossbreed (n = 11), Labrador retriever (n = 11), English springer spaniel (n = 10), boxer (n = 9), and Staffordshire bull terrier (n = 4) were the most prevalent. The prevalence of discospondylitis was significantly higher in both French and English bulldogs compared with the dogs of “other” breeds (both *P* < .001). This equated to a 3.4 (1.6‐6.7, 95% CI) times higher prevalence in French bulldogs and 4.3 (1.7‐9.8, 95% CI) times higher in English bulldogs, compared with the general hospital cohort. No statistically significant difference in prevalence was evident between French (0.48%) and English (0.60%) bulldogs (*P* = .63).

The median age at presentation was 1.1 years in French bulldogs (range, 0.5‐9.2 years), 1.0 years in English bulldogs (range, 0.4‐7.0 years), and 7.3 years in “other” breeds (range, 0.3‐14.0 years). French and English bulldogs were significantly younger at presentation compared with “other” breed dogs (both *P* < .001). Age at presentation was not significantly different between French (1.1) and English (1.0) bulldogs (*P* = .87).

In total, 118 IVDs were affected by discospondylitis with the majority of dogs having a single affected IVD (n = 101; 93.5%), whereas 2 IVDs were affected in 2 French bulldogs and 2 “other” breed dogs, and 3 IVDs were affected in 1 English bulldog and 2 “other" breed dogs. Overall, discospondylitis was diagnosed at 15 IVDs in French bulldogs and 10 IVDs in English bulldogs. The most common site of discospondylitis was L7‐S1 (n = 53; 44.9%), followed by L1‐L2 (n = 7; 5.9%); T8‐T9, T9‐T10, T10‐T11, T11‐T12, T13‐L1, and L3‐L4 (all n = 5, 4.2%); C6‐C7, T4‐T5, T5‐T6, and T12‐T13 (all n = 4; 3.4%); L2‐L3 (n = 3; 2.5%); C4‐C5, C7‐T1, and T7‐T8 (all n = 2; 1.7%); C3‐C4, T3‐T4, and L4‐L5 (all n = 1; 0.85%). The frequency of discospondylitis diagnosed within each vertebral column region (cervical, thoracolumbar, and lumbosacral) is summarized in Table 1 along with key clinical features. No statistical difference was evident between discospondylitis location in French and English bulldogs and dogs of “other” breeds (*P* = .67).

**TABLE 1:** Summary of clinical presentation and diagnostic imaging findings in dogs diagnosed with discospondylitis.

	Dogs with discospondylitis	Total hospital cohort	Age (years), median (range)	>1 CVM present in vertebral column	Total intervertebral disc spaces with discospondylitis	Vertebral region of discospondylitis intervertebral disc space			CVM at discospondylitis location	Discospondylitis at location of maximal kyphosis
						Cervical	Thoracolumbar	Lumbosacral		
French bulldog	13	2690 (0.48%)	1.1 (0.5–9.2)	12 (92.3%)	15	0 (0.0%)	11 (73.3%)	4 (26.7%)	12 (80.0%)	6 (40.0%)
English bulldog	8	1330 (0.60%)	1.0 (0.4–7.0)	6 (75.0%)	10	0 (0.0%)	5 (50.0%)	5 (50.0%)	5 (50.0%)	4 (40.0%)
Other breeds	87	74 046 (0.12%)	7.3 (0.3–14.0)	1 (1.1%)	93	9 (9.7%)	40 (43.0%)	44 (47.3%)	1 (1.1%)	0 (0.0%)

Abbreviation: CVM, congenital vertebral body malformation.

Congenital vertebral body malformations were identified in 12 (92%) French bulldogs, 6 (75%) English bulldogs, and 1 (1.1%) “other” breed dog. When present, multiple vertebral body malformations were commonly identified within the midcaudal thoracic region (T5‐T12). Of the 15 IVDs diagnosed with discospondylitis in French bulldogs, 12 (80%) were adjacent to a congenital vertebral body malformation. In English bulldogs, 5 (50%) discospondylitis affected IVDs were adjacent to vertebral body malformations, from a total of 10 affected IVD. Discospondylitis was not diagnosed adjacent to a congenital vertebral body malformation in any “other” breed dogs. A statistically significant difference was identified between the presence of vertebral body malformations adjacent to IVDs diagnosed with discospondylitis in French and English bulldogs compared with “other” breeds (*P* < 0.001). Discospondylitis was diagnosed at the IVD of maximal kyphosis within the vertebral canal in 6 (40%) French bulldogs and 4 (40%) English bulldogs, respectively. Vertebral malformations identified at the site of discospondylitis included: ventral and median aplasia (butterfly vertebrae; n = 8), ventral aplasia (dorsal hemivertebra; n = 7), ventral hypoplasia (ventral wedge‐shape; n = 2), and L7 transitional vertebra (n = 1). Discospondylitis was exclusively associated with ventral and median aplasia (butterfly vertebrae) within English bulldogs. Vertebral body malformations were evident at both adjacent vertebrae to the discospondylitis affected IVD in 3 dogs.

## 4 DISCUSSION

In this study, we report the prevalence of discospondylitis in French and English bulldogs within a referral hospital cohort and investigated for possible associations with congenital vertebral body malformations and vertebral column kyphosis. Our results revealed a significantly higher prevalence of discospondylitis diagnosis within French and English bulldogs compared with the “other” breed cohort. The prevalence of discospondylitis was found to be 3.4 times higher in French bulldogs and 4.3 times higher in English bulldogs, compared with other breeds. No pugs were diagnosed with discospondylitis at either institution during the study period, despite being presented in similar overall numbers to both French and English bulldogs. Despite similar brachycephalic phenotypic characteristics with bulldogs, Pugs do not have the truncated *DISHEVELED 2* and are therefore not considered a “screw‐tailed” brachycephalic breed.^19^ As a result, congenital vertebral body malformations are less frequently identified within the pug breed, compared with French and English bulldogs, which could offer an explanation for apparent difference in predisposition for discospondylitis between the breeds.^13^, ^20^ Within the “other” breed dogs, Labrador retrievers, boxer, and crossbreeds were over‐represented, consistent with the findings of previous studies.^5^, ^12^

French and English bulldogs diagnosed with discospondylitis were significantly younger, often being affected around 1 year of age (median of 1.1 and 1.0 years of age, respectively). Conversely, the “other” breed dogs were commonly middle‐aged (median 7.3 years), consistent with the age at diagnosis reported by previous large studies.^5^ The young age of onset, is remarkably similar to the typical age for the development of myelopathic neurological signs associated with congenital vertebral body malformations in “screw‐tailed” brachycephalic breeds, with the majority reported to be affected in the 1st year of life.^27^, ^28^ Discospondylitis should therefore be considered a potential differential diagnosis in young “screw‐tailed” brachycephalic dogs presenting with signs of spinal hyperaesthesia, myelopathy, or lameness. Although it is currently unknown why French and English bulldogs are affected at such a young age, it is possible that the pathophysiology of discospondylitis differs between “screw‐tailed” brachycephalic breeds and other breeds.

The underlying etiology of congenital vertebral body malformations remains poorly defined, with congenital changes to vertebral vascularization, genetic defects, and insult to cartilaginous proliferation previously speculated.^15^, ^18^, ^29^, ^30^ Dysplasia of the vertebral vascular canal, characterized by variable defects of ossification in the region of the vascular canal, has been recently reported in French and English bulldogs.^31^ Slow or turbulent blood flow through the vascular channels of the vertebral endplate are suspected to predispose to infection from hematogenous sources.^1^ It could, therefore, be hypothesized that congenital vertebral body malformations are associated with alterations in vertebral vascularization. Altered or aberrant vascularization could consequently result in a decreased blood flow velocity or increased blood transit time allowing for a greater chance of colonization by blood‐borne bacteria. Studies anatomically assessing the vascularization of vertebral body malformations are currently lacking within the veterinary and human literature. Histopathological analysis of the vertebral body malformations and their associated vascularization would be required to confirm or refute this hypothesis. Vertebral body malformations are associated with accelerated degeneration of the IVD.^26^ In human medicine, degeneration of the IVD has been correlated with concomitant changes to the vertebral endplate.^32^ The precise nature of the causal relationship between IVD degeneration and pathomorphological changes to the endplate is yet to be fully understood.^33^ Subsequent biochemical and morphological changes to the bone of the vertebral endplate could increase the risk of establishment of infection from hematogenous sources. The potential contribution of altered forces resulting from changes in gait associated with vertebral malformations cannot be discounted.^24^

Despite congenital vertebral body malformations being commonly identified within “screw‐tailed” brachycephalic dogs, they are seldom the primary cause for signs of neurological disease.^20^, ^21^ Overall, 80.7% of neurologically normal “screw‐tailed” brachycephalic dogs have at least 1 vertebral body malformation, whereas multiple thoracic malformations are identified in 59.6% to 64.2%.^13^, ^29^ Within this study, discospondylitis was significantly associated with the presence of a congenital vertebral body malformation adjacent to the affected IVD, occurring in 80% and 50% of affected IVDs in French and English bulldogs, respectively. Discospondylitis was found to be most commonly associated with the ventral and median aplasia (butterfly vertebrae) in English bulldogs and ventral aplasia (dorsal hemivertebra) subtypes in French bulldogs. These vertebral body malformations are considered the most common subtypes within French and English bulldogs and would therefore be expected to be encountered frequently.^29^, ^34^ The association of discospondylitis with vertebral body malformation subtype should therefore be interpreted with caution.

Being retrospective in nature, this study has several limitations. Despite being multi‐institutional, the study relies upon a relatively small number of French and English bulldogs diagnosed with discospondylitis. This could lead to type II statistical error and therefore restrict the conclusions that can be made. Although discospondylitis was found to be commonly associated with IVDs adjacent to congenital vertebral body malformations, the high prevalence of malformations in “screw‐tailed” brachycephalic dogs makes it challenging to definitively conclude a causal relationship, rather than being the result of association. A subjective assessment of the imaging studies was used to assess for the location of maximal kyphosis of the vertebral column. Use of the modified Cobb method would have allowed for an objective measurement of the degree of vertebral kyphosis. The diagnosis of discospondylitis was based upon the widely reported clinical features and diagnostic imaging criteria which would be expected to be robust; however, histopathological confirmation was not required. Case follow‐up was beyond the scope of this study, and therefore, further studies are needed to evaluate the implication of concurrent congenital vertebral body malformations on the prognosis, response to treatment, or incidence of relapse of discospondylitis.

## 5 CONCLUSIONS

The prevalence of discospondylitis was 3.4 times higher in French bulldogs and 4.3 times higher in English bulldogs, compared with the general hospital cohort. Although congenital vertebral body malformations are commonly interpreted as incidental diagnostic findings, they were frequently found to be associated with discospondylitis in both French and English bulldogs. Furthermore, French and English bulldogs were significantly younger when diagnosed with discospondylitis and commonly had clinical signs within the 1st year of their life.

## CONFLICT OF INTEREST DECLARATION

Authors declare no conflict of interest.

## OFF‐LABEL ANTIMICROBIAL DECLARATION

Authors declare no off‐label use of antimicrobials.

## INSTITUTIONAL ANIMAL CARE AND USE COMMITTEE (IACUC) OR OTHER APPROVAL DECLARATION

Approved by the Royal Veterinary College Social Sciences Research Ethical Review Board (RVC; SR2022‐0002).

## HUMAN ETHICS APPROVAL DECLARATION

Authors declare human ethics approval was not needed for this study.
